# Thermal and Rheological Properties of Gluten-Free, Starch-Based Model Systems Modified by Hydrocolloids

**DOI:** 10.3390/polym14163242

**Published:** 2022-08-09

**Authors:** Polona Megusar, David Stopar, Natasa Poklar Ulrih, Iztok Dogsa, Iztok Prislan

**Affiliations:** 1 Department of Food Science and Technology, Biotechnical Faculty, University of Ljubljana, 1000 Ljubljana, Slovenia; 2 Department of Microbiology, Biotechnical Faculty, University of Ljubljana, 1000 Ljubljana, Slovenia

**Keywords:** hydrocolloids, gluten-free, differential scanning calorimetry, rheology

## Abstract

Obtaining good-quality gluten-free products represents a technological challenge; thus, it is important to understand how and why the addition of hydrocolloids influences the properties of starch-based products. To obtain insight into the physicochemical changes imparted by hydrocolloids on gluten-free dough, we prepared several suspensions with different corn starch/potato starch/hydroxpropyl methyl cellulose/xanthan gum/water ratios. Properties of the prepared samples were determined by differential scanning calorimetry and rheometry. Samples with different corn/potato starch ratios exhibited different thermal properties. Xanthan gum and HPMC (hydroxypropyl methyl cellulose) exhibited a strong influence on the rheological properties of the mixtures since they increased the viscosity and elasticity. HPMC and xanthan gum increased the temperature of starch gelatinization, as well as they increased the viscoelasticity of the starch model system. Although the two hydrocolloids affected the properties of starch mixtures in the same direction, the magnitude of their effects was different. Our results indicate that water availability, which plays a crucial role in the starch gelatinization process, could be modified by adding hydrocolloids such as, hydroxypropyl methyl cellulose and xanthan gum. By adding comparatively small amounts of the studied hydrocolloids to starch, one can achieve similar thermo-mechanical effects by the addition of gluten. Understanding these effects of hydrocolloids could contribute to the development of better quality gluten-free bread with optimized ingredient content.

## 1. Introduction

Production of high quality gluten-free bread is a challenge since gluten confers unique viscoelastic properties to dough [1]. To overcome this challenge, different approaches are used in preparing gluten-free products, such as the use of different gluten-free flours (rice, maize), starches (corn, potato, cassava), and ingredients such as hydrocolloids [2,3,4,5,6,7,8]. Hydrocolloids are a class of food ingredients that are widely used in the development of food structure [9]. Generally speaking, we can define hydrocolloids most simply as water-soluble polymers that contribute viscosity and gelation in solution [10]. The hydrocolloids comprise polysaccharides and proteins of commercial importance that are added to food products to control, for example, the stability and rheological and organoleptic properties [11]. When making gluten-free products, one of the main challenges is to ensure that the product has desired texture as well as mouth feel comparable to the gluten-containing product. In comparison with gluten-containing bread, the gluten-free bread has higher staling tendency. Various studies have been conducted to improve the textural qualities of gluten-free bread using components such as starch, pectins, HPMC, and xanthan gums [2,3,4,5,6,7]. Properties of hydrocolloids might change due to interactions with other food polymers and components [12]. Due to complex nature of polymer interactions, there is currently no reliable method to predict the outcome of such interactions on either the quality of dough or final baked product.

There is an increasing interest for gluten-free products in the market. People who suffer from celiac disorder avoid the gluten-containing products. Currently, gluten-free diet is the only treatment for these disorders. Gluten is important to retain gas and to obtain the desired volume, and texture of a dough system [13]. It is essential to form a strong protein network required for the desired viscoelasticity. Other materials, such as hydrocolloids, that mimic viscoelastic properties of gluten and increase the dough’s gas-retaining ability are used as replacements in gluten-free bakery products [1,7,14,15,16]. Hydrocolloids are hydroxyl group (-OH) rich molecules that can form hydrogen bonds with water molecules [11]. They improve texture, increase moisture content, increase the dough’s gas-retaining ability by strengthening the boundaries of gas cells, modify viscosity, and improve the overall quality of the bread [17]. Hydrocolloids are capable of forming a stable polymeric network that entraps granules of starch and delays the release of amylose [18,19]. Some of the most commonly used hydrocolloids are hydroxypropyl methylcellulose (HPMC) and xanthan gum [1,11,17,20,21,22]. HPMC acts as a bread improver since it contributes to a higher volume of the bread and increases the shelf life due to its water-retaining ability [14,23]. It inhibits moisture migration towards the bread surface and thus slows its stalling process during storage [5,24,25,26]. The addition of xanthan gum results in high apparent viscosity, but the final bread exhibits lower specific volume [14]. In general, the interactions among different hydrocolloids can be well studied through determination of their viscoelastic properties by rotational rheology [27,28,29,30,31].

Starches are usually considered gelling materials and they significantly contribute to the texture, appearance, and overall acceptability of cereal-based foods [32,33,34]. During the bread baking process, starch granules gelatinize, i.e., they swell and are partially solubilized [35]. This occurs above a characteristic temperature known as the gelatinization temperature, which in most starches is between 60 and 80 °C [36]. During gelatinization, the amylose is dissolved and progressively released from the granules. This process consists of two steps: (1) hydration or diffusion of the solvent through the granule and (2) melting of the starch crystallites. Amylose content and starch granules size are two of the most important factors why the temperature and enthalpy of gelatinization differ with respect to starch types. Temperature and enthalpy of gelatinization can be studied by thermal techniques such as differential scanning calorimetry (DSC) [37,38]. Higher starch gelatinization temperatures lead to higher final bread volume, because the change from dough, a fluid-aerated emulsion, to a solid porous structure takes place later and allows a longer time to increase the volume [39]. On the other hand, in the process of formation of starch-based products, the rheological properties of starch play a significant role, especially in the steps prior to the heat treatment. For example, the kneading and shaping of the dough are typically performed at room temperature, where no gelatinization takes place. The rheological methods, such as rotational rheometry, can provide an inside into polymer interactions under such conditions.

The objective of this study was to explore the effects of three different polysaccharides (HPMC, xanthan gum, and starch (either potato or corn starch)) on the thermal and rheological properties of the model gluten-free systems. Several model system formulations were prepared where ingredients were studied separately and together to better understand their role in the making of gluten-free bread.

## 2. Materials and Methods

### 2.1. Composition of Model Systems

The model system formulations contained corn starch (Agrana, Gliesdorf, Austria), potato starch (Helios d.o.o., Domžale, Slovenia), hydroxypropyl methylcellulose, HPMC (Dow Europe GmbH, Horgen, Switzerland), wheat gluten (Žito, d.d., Ljubljana, Slovenia), and xanthan gum (Neimenggu Fufeng Biotechnologies, Huhehaote, China).

### 2.2. Water Content of Model System Ingredients

Weight loss properties of all samples were characterized using TGA 55 (TA Instruments, New Castle, DE, USA). The samples were first heated from 25 to 105 °C at the rate of 20 °C/min and weight loss was followed isothermally for another 30 min. The total masses of water lost were as follows: 11.2% for corn starch, 13.1% for potato starch, 5.3% for HPMC, 7.3% for gluten, and 14.5% for xanthan gum.

### 2.3. Starch Suspensions Preparation

The 20%, 30%, 40%, 50%, and 60% (*w*/*w*) suspensions of corn or potato starch were prepared in water. The 40% and 60% (*w*/*w*) corn starch suspensions were prepared in 30% (*v*/*v*) ethylene glycol (Merck, Darmstadt, Germany). Powdered starch was weighed and mixed before adding water or 30% ethylene glycol. After addition of water/30% ethylene glycol, the sample was stirred for 1 min on a vortex stirrer (Vibromix 104 EV, Tehtnica, Železniki, Slovenia) at maximum speed. The sample analysis (Differential Scanning Calorimetry (DSC), Rheology) was performed immediately after preparation.

### 2.4. Model Systems Preparation for DSC and Rheological Analysis

To prepare different model system formulations (Table 1), powdered ingredients were weighed and mixed before adding water. After addition of water, the sample was stirred for 1 min on a vortex stirrer at maximum speed. The sample analysis was performed immediately after preparation. The expressed percentages in the text of prepared solutions are mass per mass (*w*/*w*). Samples 1–9 were prepared by keeping the *w*(ingredients):*w*(water) = 1:1. Samples 10–12 were used to compare the viscoelastic properties of individual hydrocolloids.

### 2.5. DSC of Starch and Model System Suspensions

Differential Scanning Calorimetry (DSC) measurements were performed with a TA Instruments differential scanning calorimeter (model DSC 2500, New Castle, DE, USA) and the collected data were evaluated using TA instruments TRIOS software (V5.1.1.46572). Temperature and cell constant calibration of the instrument were carried out with Indium reference samples and calibration of heat capacity *C_p_* was performed with sapphire crystal, provided by TA Instruments. Samples of model system suspension (~10 mg) were placed in hermetically sealed Tzero Aluminium pans and an identical empty pan was used as a reference.

All of the samples were heated from 25 to 100 °C with a heating rate of 5 °C/min. We subtracted baselines and divided the resulting heat flow by starch mass and heating rate, thus obtaining the change in heat capacity per gram of starch as a function of temperature. The observed heat effects were characterized by calculating the change in enthalpy as the area under experimental curve and transition temperature was determined as the curve peak position. Positive changes in heat capacity correspond to endothermic processes (endo up) and negative changes in heat capacity correspond to exothermic processes (exo down).

### 2.6. Rheology of Starch and Model System Suspensions

Viscoelastic properties of samples were determined through amplitude sweep tests measured on an Anton Paar Physica MCR 302 rotational rheometer (Anton Paar, Graz, Austria) operated by Rheoplus/32 V3.62 software. We used the plate–plate system (PP25) with a plate diameter of 25 mm. The gap between the plates during measurements was set to 1 mm. The temperature was kept at (20.00 ± 0.01) °C. In these measurements, the shear stress response to an applied shear strain was measured. The ratio of the shear stress to shear strain is important and is called the complex modulus, G*. This modulus is comprised of the storage modulus (elastic part), *G*′ and loss modulus (viscous part), *G*″ [40]. The dynamic oscillatory testing was conducted with oscillatory amplitude strain sweep test from 0.01% to 1000% in 25 logarithmically spaced steps, at a constant frequency of 10 Hz. The measurement time at each strain was automatically adjusted by the software. Direct stress oscillation (DSO) was set to “Auto”. In this way, the storage (*G*′) and loss modulus (*G*″) were measured as a function of shear strain amplitude.

### 2.7. The Size and Molecular Weight of Starch and Hydrocolloids

The HPMC used was labelled as K99, food grade. According to the producer specifications, K99 has M_w_ = (100 ± 10) kDa, polydispersity index ~4, methoxyl and hydroxypropyl substitution (*w*/*w*) 21.5% ± 2.5% and 9.5% ± 2.5%. To obtain the Mw of xanthan gum, we determined its intrinsic viscosity in 0.1 M NaCl [41,42]. A cone plate measuring system was used (CP50-1) with plate a diameter of 50 mm. The temperature was kept at (20.00 ± 0.01) °C. The viscosity as a function of shear rate was determined in the range from 200 to 2000 s^−1^. Several xanthan gum concentrations in the range from 0.01 g/dL to 0.03 g/dL were measured and for specific viscosity calculation, the average viscosity from the non-pseudoplastic part (1000 to 2000 s^−1^) of the flow-curves was taken into account. From the obtained intrinsic viscosity, [η] = (29 ± 3) dL/g, we estimated the M_W_ of xanthan gum by the Mark–Houwink relation, with α = 1.14 and K = 1.7 × 10^−6^ [41,42] to be (2200 ± 300) kDa. To characterize the size of starch granules, we used light microscopy. First, we prepared 1% (*w*/*v*) suspensions that were stabilized in 0.1% (*w*/*v*) xanthan gum to reduce weak aggregation of granules that we observed in water-only suspensions. Approx. 5 µL of suspension was transferred to glass slide and covered by cover slip. Using the microscope Zeiss Axio Observer Z1 with 10×/0.3 objective, operating in bright field mode and equipped with an AxioCam MRm Rev.3 camera, we took at least 25 view fields of starch suspensions for each of three replicates. Using Fiji-ImageJ tools, we obtained a Feret diameter (the longest distance between any two points in the granule) of more than 2000 granules. The parameters of the obtained distribution were as follows. The mean diameter of potato starch granules was (24 ± 6) µm with a standard deviation of (17 ± 1) µm and the mean diameter of corn starch granules was (13 ± 2) µm with a standard deviation of (8 ± 1) µm.

### 2.8. Data Analysis

All samples were prepared and measured at least in triplicates. The calculated mean values and standard deviations are shown in figures unless stated otherwise. Loss factor (tan δ), which is a ratio of loss and storage modulus, was determined in linear viscoelastic range of the two moduli. Unpaired Student’s T-test, two-sided, was used to calculate the statistical significance of data sets. A *p* value of less than 0.05 was considered statistically significant.

## 3. Results and Discussion

### 3.1. Starch Gelatinization

**Figure 1:**
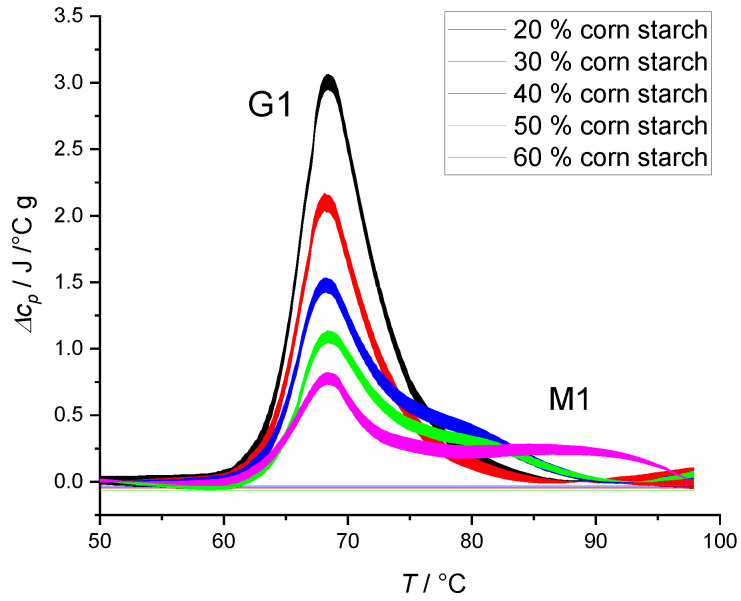
DSC thermograms of aqueous suspensions with different corn starch contents. The sets of experiments were performed by increasing starch concentration from 20% to 60% (*w*/*w*). The lines represent averages of three independent experiments (*n* = 3) and the line thickness corresponds to SD.

The gelatinization of starch is an important event during baking of the bread when dough is exposed to elevated temperatures. Heating an aqueous starch suspension induces a number of structural changes in granules during gelatinization. Thermally induced structural changes depend on the amount of water in starch suspension [43,44,45]. Our investigation began by studying the simplest system, i.e., starch solution without any added hydrocolloids. Figure 1 shows thermograms of different starch weight fractions to water suspensions. When starch suspensions were heated in the presence of excess water, a single symmetric endothermic transition, usually denoted as G1, was observed in the lower temperature region (Figure 1; 20 and 30% *w*/*w* corn starch suspension). As the amount of available water decreased, a biphasic endothermic transition, with tailing shoulder, usually denoted asM1, was observed (Figure 1; 40–60% *w*/*w* corn starch suspensions). As the water level was reduced, the height of the first peak diminished and consequently enthalpy of transition, Δ*H*_tr_, was reduced (Table 2). The transition temperature of the first peak did not shift with increasing starch weight fraction and stayed at *T*_tr_ ≈ 68 °C.

There are a few possible explanations for this phenomenon. Donovan’s model from 1979 explains G1 and M1 endotherms in terms of water availability in amorphous and crystallite regions [46]. Gelatinization starts in amorphous regions of the granule, which can be associated with the first transition (G1), while the tailing shoulder (M1) represents the melting of the remaining less-hydrated crystallites. A second possible explanation was given by Evans and Haisman in 1982 [47]. The successive gelatinization peaks were suggested to reflect the melting of the crystallites with different stabilities due to a gradient of water within the sample. Slade’s and Levine’s model from 1988 proposes that the G1 endotherm reflects primarily plasticization in amorphous regions, whereas the M1 endotherm reflects non-equilibrium melting of crystallites [48]. In the year 2000 Waigh, Gidley, Komanshek, and Donald proposed a new mechanism, focusing on the change in the crystalline structure during gelatinization [49]. The G1 endotherm is considered to reflect the helix–helix dissociation whereas the M1 endotherm is a result of the helix–coil transition of amylopectin chains [38,45,50].

**Figure 2:**
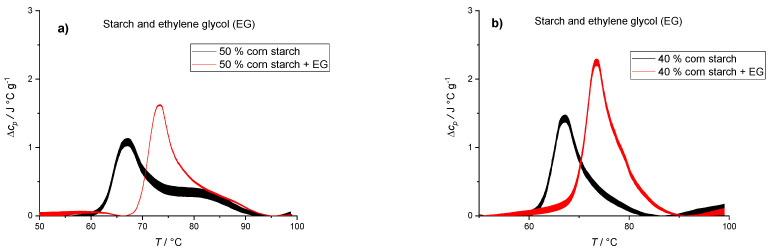
DSC thermograms of starch suspensions in water and 30% ethylene glycol (EG). (**a**) 50% (*w*/*w*) corn starch solution in water (blackline) and in 30% EG solution (red line) (**b**) 40% (*w*/*w*) corn starch suspensions in water (black line) and in 30% EG solution (red line). The lines represent averages of three independent experiments (*n* = 3) and the line thickness corresponds to SD.

The results in Figure 1 suggest that water availability plays a crucial role in the gelatinization process. To further investigate the role of water, we prepared and analyzed starch suspensions in 30% ethylene glycol (EG) solution. The water activity of 30% EG solution was lower compared [51] with pure water and thus should affect the gelatinization process. Replacing water with 30% EG resulted in a shift of the first thermally induced transition (G1) by 6 °C (Figure 2). On the other hand, replacing water with 30% EG did not seem to affect the position of the second transition (M1). Position shift of the first peak was not observed when amount of water in starch suspension was lowered (Figure 1), and hence gelatinization of starch can also be governed by solvent properties other than the starch:water ratio. When small solutes are added to water, they change its thermodynamic properties, affecting the water’s ability to interact with other components in the system. Since the reactivity of water is lowered, the chemical and physical changes involving water will require more energy [52]. Besides interacting with water, ethylene glycol can have a stabilizing effect by H-bonding, which enhances the strength of starch granule. Consequently, a shift of thermally induced transition to higher temperatures can be observed (Table 3). Albeit within the experimental error, the data also suggest an increase in enthalpy after the addition of EG for 40% and 50% corn starch. Additionally, Table 3 confirms that by decreasing the concentration of starch, more solvent molecules (water and ethylene glycol) are available, leading to a higher degree of gelatinization and an increase in enthalpy in both water and EG starch suspensions.

**Figure 3:**
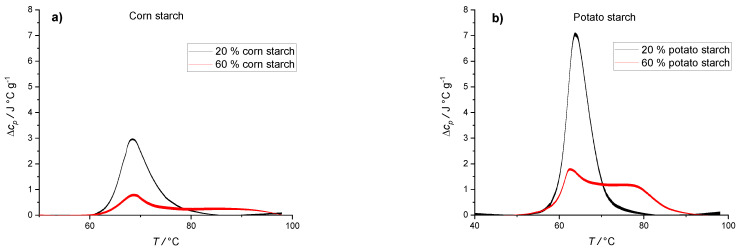
DSC thermograms of corn and potato starch aqueous suspensions at different starch:water ratios. (**a**) DSC thermogram of corn starch suspension; 20% (*w*/*w*) starch in water (black line) and 60% (*w*/*w*) suspension of starch in water (red line). (**b**) DSC thermogram of potato starch suspension; 20% of starch in water (black line) and 60% of starch in water (red line). The lines represent averages of three independent experiments (*n* = 3) and the line thickness corresponds to SD.

The water content and activity are, however, not the only factors affecting gelatinization process. As can be seen from Figure 3, the heating thermograms of corn starch suspensions (Figure 3a) and potato starch suspensions (Figure 3b) markedly depend on the starch type used. Additionally, corn starch suspensions exhibited higher transition temperatures, *T*_tr_, and lower transition enthalpies, Δ*H*_tr_ (*T*_tr_ (20% corn starch) = 67.0 °C, Δ*H*_tr_ (20% corn starch) = 22.3 J/g), than potato starch suspensions (*T*_tr_ (20% potato starch) = 63.9 °C, Δ*H*_tr_ (20% potato starch) = 49.4 J/g). This can be explained by different sizes of starch granules and amylose content. Potato starch granules are on average bigger than corn starch granules and tend to absorb higher amount of water during gelatinization [53]. Consistent with the literature [53,54], the mean diameter of potato starch granules in our experiments was (24 ± 6) µm. On the other hand, the diameter of corn starch granules was much smaller (13 ± 2) µm. The average amylose content of native potato starch is around 28%, whereas the average amylose content in corn starch is lower and around 22%. Higher amylose content shifts the temperature of transition to lower *T*_tr_ values and higher Δ*H*_tr_ values [53]. Because corn starch granules are smaller (absorb less water) and have lower amylose content, gelatinization of corn starch is accompanied by higher *T*_tr_ values and lower Δ*H*_tr_ values [53,54,55].

### 3.2. Effects of Hydrocolloids on a Gelatinization Behavior of Starches

**Figure 4:**
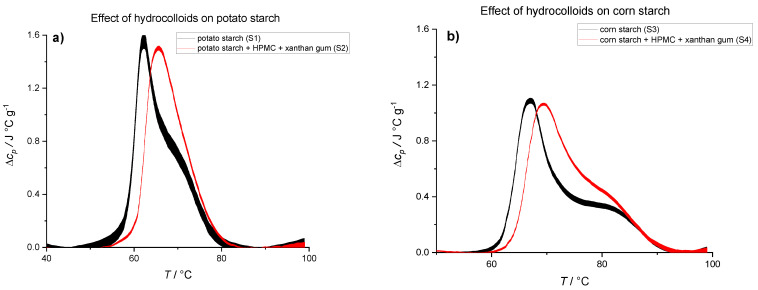
DSC thermograms showing effects of hydrocolloids on the gelatinization behavior of starches. (**a**) Thermally induced transition of 50% potato starch suspension in water (black line, S1) and thermally induced transition of 38% potato starch, 8% HPMC, and 4% xanthan gum suspension in water (red line, S2). (**b**) Thermally induced transition of 50% corn starch suspension in water (black line, S3) and thermally induced transition of 38% corn starch, *HC mixture* (8% HPMC and 4% xanthan gum) dissolved in water (red line, S4). The lines represent averages of three independent experiments (*n* = 3) and the line thickness corresponds to SD. All concentrations are given as (*w*/*w*).

Effects of hydrocolloids on gelatinization behavior of starches were investigated by adding HPMC and xanthan gum to potato (Figure 4a) and corn (Figure 4b) starch suspensions. HPMC and xanthan gum are common additives in gluten-free bakery products and very often used together, so we explored their combined effect on starch gelatinization. As a starting point, we used a HPMC:xanthan ratio which yields good quality gluten-free bread recommended by experts in the bakery industry (Žito d.d, Slovenia) and will be referred to as *HC mixture* (4% xanthan + 8% HPMC). Adding HPMC and xanthan gum to potato starch suspension (S2) resulted in an increase of transition temperature from 62.3 to 66.1 °C. A similar effect was observed when *HC mixture* was added to corn starch suspension (S4) with an increase in transition temperature from 67.4 to 70.2 °C (Table 4).

In general, polymers with a molar mass greater than ~1000 g/mol, including HPMC and xanthan, are unable to enter starch granules [56,57]. Thus, any starch gelatinization changes imposed by addition of HPMC or xanthan are due to surface interactions of hydrocolloids with starch and/or immobilization of water. Hydrocolloids are hydrophilic and change the solvent properties, interacting with starch granules and hence influencing starch swelling and gelatinization [14,58,59]. The effect of water immobilization on starch gelatinization can be observed in Figure 1 where the position of first peak did not shift but the enthalpy of transition was reduced as the starch:water ratio was increased. Figure 4 shows a different thermal behavior of starch with *HC mixture* where we can observe a shift of the first peak to higher temperatures and an increase, albeit statistically insignificant (*p* > 0.05), in transition enthalpy (Table 4). It seems that besides water retention properties, HPMC and xanthan gum have additional properties such as interfacial activity and forming gel networks on heating. Consequently, more thermal energy is required to achieve starch transformation, which is reflected by higher transition temperature. It is worth mentioning that the position of the second peak was not shifted upon the addition of *HC mixture*, thus showing similar behavior as in the case of 30% EG suspensions.

Table 4 shows that addition of hydrocolloids shifts transition temperatures for both starches to higher values. This can be attributed to the interactions of hydrocolloids with water and lower mobility of water. Besides, hydrocolloids and starch could interact and form stable complexes, which could shift transition temperatures to higher values [14,58]. Strength and type of interactions should depend on type of hydrocolloids used in suspension; therefore, we expect that changes in starch gelatinization behavior depend on the type and the amount of different hydrocolloids added to the suspension.

**Figure 5:**
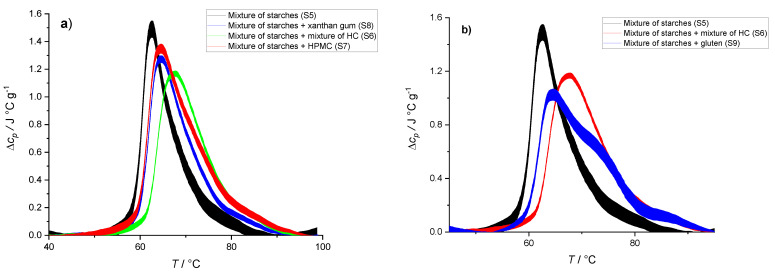
DSC thermograms of different mixtures of starch (potato and corn starch in ratio 85:15), xanthan gum, and HPMC in water. (**a**) Thermograms of 50% suspension of potato and corn starch mixture in 85:15 ratio (black line, S5), 38% suspension of starch mixture with *HC mixture* (4% xanthan gum and 8% HPMC) (green line, S6), 42% suspension of starch mixture with 8% HPMC (red line, S7), and 45% suspension of starch mixture with 5% xanthan gum (blue line, S8). (**b**) Thermograms of 50% suspension of potato and corn starch mixture in 85:15 ratio (black line, S5), 38% suspension of starch mixture with *HC mixture* (4% xanthan gum and 8% HPMC) (red line, S6), and 38% suspension of starch mixture with 12% gluten (blue line, S9). The lines represent averages of three independent experiments (*n* = 3) and the line thickness corresponds to SD. All concentrations are given as (*w*/*w*).

In order to investigate the individual and synergistic effects of HPMC and xanthan gum on starch gelatinization, we performed a series of experiments where HPMC and xanthan gum were added to a mixture of corn and potato starch suspension. The experimentally tested mixtures of potato and corn starch were mixtures commonly used in baking industry for gluten-free products. The ratio of potato to corn starch commonly used in the baking industry (*m*(corn starch):*m*(potato starch) = 85:15) was chosen. The thermograms (Figure 5) show the effect of adding xanthan gum and/or HPMC to the starch mixture (samples S5, S6, S7, and S8). Adding either HPMC or xanthan gum to the starch mixture suspensions resulted in similar changes of thermally induced starch gelatinization behavior (Figure 5a). Addition of hydrocolloids shifted the transition temperature to higher temperatures (Δ*T*_tr_(xanthan) = 2.3 °C and Δ*T*_tr_(HPMC) = 2.4 °C) when compared with the transition temperature of the starch mixture suspension without the addition of hydrocolloids. From the data in Table 5, we can calculate an increase in enthalpy when xanthan gum (ΔΔ*H*_tr_(xanthan) = 0.3 J/g) or HPMC (ΔΔ*H*_tr_(HPMC) = 3.5 J/g) was added to the starch mixture. Unpaired *t*-test showed that there was a significant difference (*p* < 0.05) between transition enthalpies of starch suspension and transition enthalpies of starch suspension with HPMC; thus, only ΔΔ*H*_tr_(HPMC) is statistically significant. Since the mass ratio of HPMC is twice that of xanthan gum in the starch suspension (8 vs. 4% *w*/*w*), higher transition enthalpy of the starch suspension with HPMC could correspond to a higher amount of hydrocolloid. On the other hand, HPMC is less hydrophilic than xanthan gum due to its hydrophobic methoxy side groups and bulkier due to its hydroxypropyl side groups [22]. Because of these properties, HPMC can interact differently with water and starch, leading to higher enthalpy of starch gelatinization. When both hydrocolloids were added to the starch suspension at the same time, the shift in temperature was even greater (Δ*T*_tr_(*HC mixture*) = 5.9 °C), whereas an increase in enthalpy was lower than upon addition of only HPMC and similar as upon addition of xanthan gum (ΔΔ*H*_tr_(*HC mixture*) = 0.7 J/g). This result suggests that HPMC and xanthan gum may interact with each other and not only with starch and water.

In order to compare the gluten-free model system with the gluten-containing system, we replaced *HC mixture* with gluten (12% *w*/*w*) (S9). Figure 5b shows the thermal behavior of such mixture compared to the thermal behavior of gluten-free starch suspension (S5) and gluten-free model systems with *HC mixture* (S6). Two-state transition can be observed during heating of starch mixture with the highest thermogram peak at *T* = 62.5 °C (Figure 5b, black line, S5) and three-state transition can be observed during heating of the starch mixture with gluten with the highest thermogram peak at *T* = 64.5 °C (Figure 5b, blue line, S9). It can be seen from Figure 5b that gelatinization in starch mixture with gluten takes place over a larger temperature region compared with the starch mixture. Additionally, data in Table 5 suggests that transition enthalpy of the starch mixture with gluten is higher than transition enthalpy of the starch mixture (ΔΔ*H*_tr_(gluten) = 1.8 J/g). Unpaired *t*-test showed that there is no significant difference (*p* = 0.13 > 0.05) between transition enthalpies of starch suspension and transition enthalpies of the starch suspension with gluten, and thus the ΔΔ*H*_tr_(gluten) value should be used cautiously. Increasing the temperature of starch gelatinization is important because higher starch gelatinization temperatures lead to higher final bread volume. If the transformation of dough from fluid aerated emulsion to a solid porous structure takes place at higher temperatures, this gives more time for the dough volume to increase, which in turn has a beneficial effect on bread quality [39,60,61]. Similar to the role of gluten in starch suspension thermal properties, the addition of all types of hydrocolloids shifts the temperature of the gelatinization to higher temperatures. Additionally, it can be observed that after the addition of xanthan and HPMC, gelatinization takes place over a wider temperature range and over multiple transition events (Figure 5). This shows that the thermal properties of starch suspensions with gluten can be mimicked by replacing gluten with hydrocolloids.

### 3.3. Effects of Hydrocolloids on Rheological Properties of Starch

**Figure 6:**
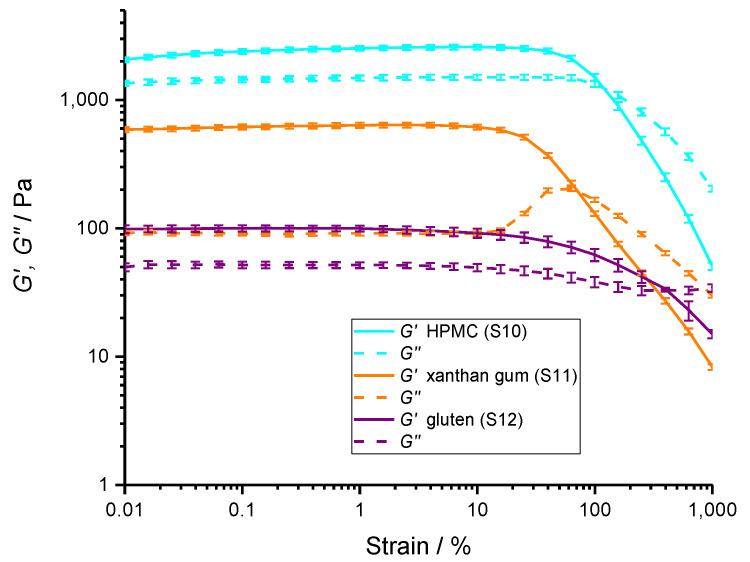
Rheology: Oscillatory amplitude tests performed on samples of individual hydrocolloids aqueous solutions. Storage (*G*′, -----) and loss modulus (*G*″, - - - -) of 8% xanthan gum in water (orange line, S11), 8% HPMC in water (blue line, S10) and 8% gluten in water (purple line, S12). The lines represent averages of three independent experiments (*n* = 3) and the error bars correspond to SD. All concentrations are given as (*w*/*w*).

**Figure 7:**
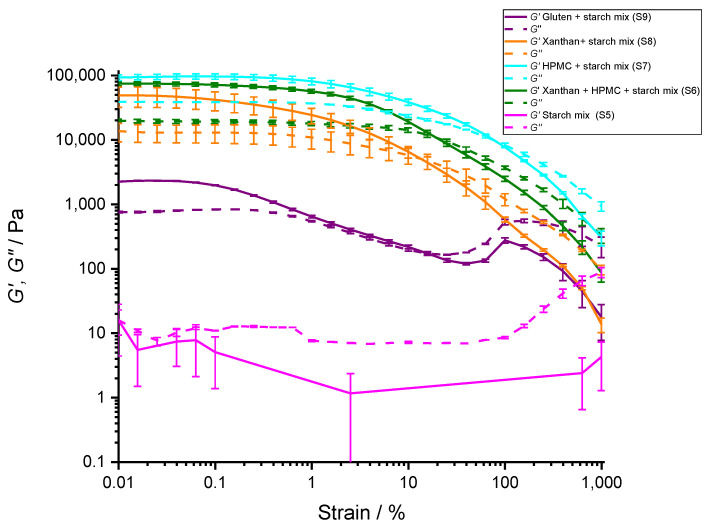
Rheology: Oscillatory amplitude tests performed on samples of starch/hydrocolloids mixtures. To all samples the water was added in a ratio 1:1, water:dry ingredients. Storage (*G*′, -----) and loss modulus (*G*″, - - - -) of 12% gluten, 38% starch mixture and water (purple line, S9), 4% xanthan gum and 46% starch mixture (orange line, S8), 8% HPMC and 42% starch mixture (blue line, S7), and 8% HPMC and 4% xanthan gum, 38% starch mixture and water (green line, S6), and starch mixture alone (pink line, S5). Starch mixture (starch mix) consists in all presented cases of 0.85 potato:0.15 corn starch. The lines represent averages of three independent experiments (*n* = 3) and the error bars correspond to SD. All concentrations are given as (*w*/*w*). Only the data above the sensitivity of the rheometer (*G* > 1 Pa) are shown.

In general, the interactions between polymer molecules manifest in the rheological properties of the mixture. Amplitude sweep measurements were performed on pure hydrocolloids (Figure 6) and different mixtures of starch and hydrocolloids (Figure 7). Only the most relevant results to compare the effects between hydrocolloids, starch, and gluten are shown.

Pure HPMC (S10), xanthan gum (S11), and gluten (S12) solutions have elastic character (*G*′ > *G*″), typical for gel-like systems and entangled polymer solutions. At the same concentrations of polymers, xanthan solution had the highest tan δ ratio (Table 6), suggesting a more solid-like behavior. In addition, xanthan, which has charged side chains, had a *G*″ peak. Song et al. (2006) also observed a *G*″ peak in less concentrated xanthan solutions (i.e., 1% to 4%) [62]. *G*″ peaks are typically absent in linear unlinked polymers and may occur due to the relative motion between the polymer molecules, flexible end-pieces of chains and side chains, long network bridges, mobile single particles, agglomerates, or superstructures not linked or otherwise fixed in the network [63].

As the shear strain increases, it reaches the critical value (*γ_c_*), where either *G*′ or *G*″ starts to change, marking the end of the linear viscoelastic region (LVE) region and indicating structural breakdown. The LVE was comparable for gluten and xanthan, but was much higher for HPMC. Further increase in the shear strain induced additional breaks in the gel polymer network. Eventually, at *G*′ = *G*″ (flow transition point), the samples started to behave as a liquid-like material. The difference between the shear stress at the critical point and flow point indicates how compliant the hydrocolloid structure is to shear stress. The most compliant was gluten structure, whereas xanthan and HPMC had more brittle structures. In gluten, the disintegration of the polymer network proceeded in a gradual manner and gluten retained its solid-like character at a larger span of shear stresses. If cohesive energy density (*E_c_* = ½ *G*′ × *γ_c_*^2^), a measure of structure integrity [64], is calculated, one can observe that HPMC has the highest cohesive energy density followed by xanthan and gluten. Cohesive energy density in gluten was approximately 150-fold lower than in HPMC. This is consistent with the much more compliant structure of gluten compared with HPMC hydrocolloid.

Starch suspension (S5) at room temperature had almost no elasticity and very low viscosity (Figure 7, pink line). This can be explained with the presence of starch granules and absence of starch gelatinization at room temperature. At room temperature, starch granules do not dissolve and starch behaves as a suspension of starch granules. Low viscosity of starch granule suspension is consistent with the results obtained by Montes et al. [65]. The starch granules are expected to be rigid and there are no long-range interactions between them. At large shear strains (i.e., above 100%), the viscosity of the starch suspension started to increase, which is consistent with the dilatant behavior observed in the starch granule systems [66]).

When gluten was added to starch granules suspension (S12), the *G*′ increased by an order of magnitude compared with either of the pure components. The gluten at the applied concentration, however, was unable to fully prevent the dilatation effect of the starch granules at high shear strains. Nevertheless, the flow transition point in gluten increased compared with the starch suspension. The starch–gluten mixture cohesive energy density was very low, suggesting only weak interactions between the starch granules and gluten at room temperature.

Adding xanthan to the starch granules dramatically increased stability of the mixture (S8). The increased *G*′ of the mixture (50 kPa) was a non-linear combination of the contribution of starch (0.01 kPa) and xanthan (0.6 kPa) elasticities, suggesting a strong interaction between the starch and xanthan hydrocolloids. The cohesive energy of this mixture was approximately 50-fold larger than in the case of starch–gluten mixture even though the concentration of the added xanthan was much lower. The addition of xanthan also removed the dilatant starch granule behavior at high shear strains.

When HPMC was added to the starch granules, the effect was even more pronounced than in the case of xanthan (S7). This mixture had approximately 80-fold higher cohesive energy density compared with the starch–xanthan mixture and 4500-fold higher than the starch–gluten mixture. Similar to the starch–xanthan mixture, the effect of starch dilatation at higher shear strains was absent. This mixture, however, was significantly less compliant to either starch–gluten or starch–xanthan mixtures. This suggests a strong interaction between starch and HPMC at room temperature.

The three-component mixture (8% HPMC + 4% xanthan gum + 38% starch, S6) was, in terms of cohesive energy, intermediate between the starch–xanthan and starch–HPMC mixtures. This mixture decreased the high stiffness of the starch–HPMC mixture (*G*′ was 74,000 Pa), it decreased the loss factor, and had a more compliant structure.

The data suggest that starch granules may interact with HPMC and xanthan hydrocolloids. The interaction is strong and may significantly harden the mixture (up to four orders of magnitude increase in *G*′). However, the LVE critical strain, *γ_c_*, was in all mixtures significantly reduced compared with pure HPMC, xanthan gum, and gluten solutions. Adding starch to xanthan or HPMC decreased their cohesive energy density by about two orders of magnitude. In case of gluten, the cohesive energy density was further reduced for an additional order of magnitude. Additionally, the strain at the flow transition point of mixtures was significantly reduced compared with pure components. All of this indicates that rigid starch granules, when embedded into hydrocolloid network, can stabilize such network significantly. However, the stabilization effect is only evident at rest or very low shear strains; at higher strains, the starch granules weaken the structure, making it softer. In fact, this is just what is desired in quality dough. At rest, the dough should keep its shape, but for kneading it should be more compliant. Our data indicate that by fine tuning the composition of the mixture, one could in principle not only match the rheological properties of starch–gluten mixtures, but also obtain a large spectrum of rheologically different mixtures tailored for different gluten-free products.

## 4. Conclusions

In this study, the interactions between starch and two hydrocolloids (HPMC and/or xanthan gum) were studied with differential scanning calorimetry and rheology. The DSC results indicated that water availability, which plays a crucial role in the starch gelatinization process, could be modified by adding hydrocolloids. The rheology of gluten-free model systems was significantly affected by the interactions between starch and hydrocolloids. With the addition of xanthan gum and HPMC to starch, a suspension with increased viscoelasticity and increased enthalpy and temperature of starch gel transition was formed. The results indicate that by the addition of comparatively small amounts of the studied hydrocolloids to starch, one can achieve similar thermo-mechanical effects as by the addition of gluten. The addition of two hydrocolloids impacted the thermo-mechanical properties of the suspensions in the same direction, albeit with different magnitudes. This implies that the fine-tuning of dough properties by changing the amount and ratios of added HPMC and xanthan to starch mixtures is possible and could be used to optimize the production of gluten-free products.

## Figures and Tables

**Table 1:** Different model system formulations for DSC and rheological analysis. The samples in the text are referenced according to the sample name in this table.

Sample Name *	*m* (Xanthan Gum)/g	*m* (HPMC)/g	*m* (Gluten)/g	*m* (Potato Starch)/g	*m* (Corn Starch)/g	*m* (Water)/g
S1	/	/	/	1.50	/	1.50
S2	0.12	0.24	/	1.14	/	1.50
S3	/	/	/	/	1.50	1.50
S4	0.12	0.24	/	/	1.14	1.50
S5	/	/	/	1.28	0.23	1.50
S6	0.12	0.24	/	0.97	0.17	1.50
S7	/	0.24	/	1.07	0.19	1.50
S8	0.12	/	/	1.17	0.21	1.50
S9	/	/	0.36	0.97	0.17	1.50
S10	/	0.13	/	/	/	1.50
S11	0.13	/	/	/	/	1.50
S12	/	/	0.13	/	/	1.50

* (S1) 50% potato starch suspension, (S2) 38% potato starch + 12% HC mixture, (S3) 50% corn starch suspension, (S4) 50% corn starch + 12% HC mixture, (S5) 50% starch mixture suspension, (S6) 38% starch mixture + 12% HC mixture, (S7) 42% starch mixture + 8% HPMC, (S8) 46% starch mixture + 4% xanthan gum, (S9) 38% starch mixture + 12% gluten, (S10) 8% HPMC (S11) 8% xanthan gum, (S12) 8% gluten; HC mixture = the mixture of hydrocolloids (4% HPMC and 8% xanthan gum).

**Table 2:** Thermodynamic profile (Δ*H*_tr_) of thermally induced transitions of starch suspensions with different corn starch content.

	20% Corn Starch	30% Corn Starch	40% Corn Starch	50% Corn Starch	60% Corn Starch
Δ*H*_tr_ (J/g starch)	22.3 ± 1.8	15.9 ± 1.3	14.9 ± 1.2	12.6 ± 1.0	10.4 ± 1.0

**Table 3:** Comparison of thermodynamic parameters associated with thermally induced transition of different amounts of corn starch in water and 30% ethylene glycol.

	50% Corn Starch	50% Corn Starch + EG	40% Corn Starch	40% Corn Starch + EG
Δ*H*_tr_ (J/g starch)	12.6 ± 1.0	14.2 ± 0.6	14.9 ± 1.2	18.6 ± 1.5
*T*_tr_ (°C)	68.0 ± 0.5	73.0 ± 0.5	68.0 ± 0.5	74.0 ± 0.5

**Table 4:** Thermodynamic profile *(*Δ*H*_transition_ and *T*_transition_) of thermally induced transitions of starch suspensions without/with *HC mixture* (8% HPMC and 4% xanthan gum).

	Potato Starch (S1)	Potato Starch + HC Mixture (S2)	Corn Starch (S3)	Corn Starch + HC Mixture (S4)
Δ*H*_tr_ (J/g starch)	15.7 ± 1.9	15.8 ± 0.5	12.8 ± 0.8	13.8 ± 1.0
*T*_tr_ (°C)	62.3 ± 0.5	66.1 ± 0.5	67.4 ± 0.5	70.2 ± 0.5

**Table 5:** Transition enthalpies (Δ*H*_tr_) and temperatures (*T*_tr_) obtained from the thermograms in Figure 5.

	Starch Mix (Potato:Corn = 85:15) (S5)	Starch Mix + HC Mixture (S6)	Starch Mix + % HPMC (S7)	Starch Mix + 4% Xanthan (S8)	Starch Mix + Gluten (S9)
Δ*H*_tr_ (J/g of starch)	14.7 ± 1.0	15.4 ± 1.0	18.2 ± 1.5	15.0 ± 1.2	16.5 ± 1.3
*T*_tr_ (°C)	62.5 ± 0.5	68.4 ± 0.5	64.9 ± 0.5	64.8 ± 0.5	64.6 ± 0.5

**Table 6:** The values of elastic modulus, *G*′, calculated loss factor tan δ at 0.1% strain, critical strain, *γ_c_* at the end of linear viscoleasti range (LVE) in *G*′, strain at the flow transition point, and cohesive energy density calculated as *E_c_* = ½ *G*′ × *γ_c_*^2^.

	*G*′ (KPa) (in LVE)	Loss Factor, Tan δ (in LVE)	LVE Critical Strain, *γ_c_* at *G*′ (%)	Strain at Flow Transition Point (%)	Cohesive Energy Density, *E_c_* (J m^−3^)
**8% HPMC (S10)**	2.4 ± 0.1	0.61 ± 0.01	63 ± 12	130 ± 30	500 ± 100
**8% Xanthan gum (S11)**	0.61 ± 0.03	0.15 ± 0.01	25± 5	80 ± 20	19 ± 6
**8% gluten (S12)**	0.10 ± 0.01	0.52 ± 0.01	24 ± 14	500 ± 100	3 ± 2
**8% HPMC + 42% starch mixture (S7)**	97 ± 9	0.40 ± 0.04	1.0 ± 0.1	80 ± 20	5 ± 1
**4% Xanthan gum + 46% starch mixture (S8)**	50 ± 20	0.31 ± 0.01	0.16 ± 0.03	20 ± 5	0.06 ± 0.03
**12% gluten + 38% starch mixture (S9)**	2.30 ± 0.05	0.42 ± 0.01	0.10 ± 0.03	20 ± 5	0.0011 ± 0.0005
**8% HPMC + 4% Xanthan gum + 38% starch mixture (S6)**	74 ± 6	0.27 ± 0.01	0.39 ± 0.07	20 ± 5	0.6 ± 0.2

## Data Availability

Not applicable.
